# Retraction: Klotho gene and protein in human placentas according to birth weight and gestational age

**DOI:** 10.3389/fendo.2025.1717791

**Published:** 2025-10-09

**Authors:** Frontiers Editorial Office

The journal retracts the January 15 2019 article cited above.

Following publication, concerns were raised regarding the integrity of the images in the published figures. The authors failed to provide a satisfactory explanation and raw data during the investigation, which was conducted in accordance with Frontiers’ policies. As a result, the data and conclusions of the article have been deemed unreliable and the article has been retracted.

This retraction was approved by the Chief Executive Editor of Frontiers. The authors do not agree to this retraction.

Frontiers would like to thank users of PubPeer for bringing the published article to our attention.

